# Pre- and intra-operative risk factors for re-operation after valve sparing aortic root replacement using the David technique. Results from two European centers

**DOI:** 10.3389/fcvm.2026.1881845

**Published:** 2026-08-06

**Authors:** Armin Darius Peivandi, Andreas Martens, Felix Fleißner, Ruslan Natanov, Morsi Arar, Florian Helms, Malakh Shrestha, Heike Krüger, Erik Beckmann, Arjang Ruhparwar, Alessandro Leone, Giacomo Murana, Costanza Fiaschini, Davide Pacini, Francesco Campanini

**Affiliations:** 1 Department of Cardiac Surgery, Klinikum Oldenburg, Oldenburg, Germany; 2 The Carl von Ossietzky University Oldenburg, Oldenburg, Germany; 3 Department of Cardiothoracic, Transplantation and Vascular Surgery, Hannover Medical School, Hannover, Germany; 4 Minneapolis Heart Institute, Allina Health, Minneapolis, MN, United States; 5 Division of Cardiac Surgery, Cardiac Surgery Department, IRCCS, Azienda Ospedaliero-Universitaria di Bologna, Bologna, Italy

**Keywords:** David operation, prediction, re-operation, risk score, valve sparing root replacement

## Abstract

**Background:**

This study investigates pre- and intra-operative risk factors for re-operation after valve sparing aortic root replacement using the David technique to develop a risk model.

**Methods:**

A retrospective cohort study of two European centers, comprising data from 07/1993 to 01/2025 was performed. Follow up time was 120 (IQR: 141) months. Primary outcome was aortic valve re-operation. Prediction model was developed using uni- and multivariable logistic regression analysis. A risk score was derived. Calibration in the large and slope were performed. Receiver operating characteristic (ROC) analysis allowed discrimination assessment.

**Results:**

1,074 patients were included. Male sex (OR: 1.98; 95% CI: 1.16–3.39, *p* = 0.012), age at operation (OR: 0.98; 95% CI: 0.97–0.996; *p* = 0.011), pre-operative AI grade (OR: 1.61; 95% CI: 1.21–2.14; *p* = 0.001), NYHA (OR: 1.29; 95% CI: 1.0043–1.67; *p* = 0.046), bicuspid aortic valve (OR: 2.03, 95% CI: 1.18–3.49; *p* = 0.011) and prosthesis size (OR: 0.76; 95% CI: 0.66–0.86; *p* < 0.001) remained independent predictors in multivariable analysis. The risk model showed moderate discrimination [AUC: 0.722 (95% CI: 0.677–0.766)] and good calibration in the original cohort [intercept: −0.00 (95% CI: −0.49–0.49); slope: 1.00 (95% CI: 0.75–1.26)].

**Conclusion:**

Bicuspid aortic valve, higher pre-operative AI, NYHA grade and male sex were independent risk factors for re-operation. Higher age and larger prosthesis sizes were protective factors. Further multi-center research is needed to validate and refine the prediction model.

## 1 Introduction

Valve sparing aortic root replacement, using the re-implantation technique (i.e., David procedure), is associated with excellent long-term survival rates and low rates of re-operation (1, 2).

A systematic review and meta-analysis, including 44 studies, with 7,878 patients, reported overall survival rates of 99% after 1-year and 89% after 10-years. In the same study, freedom from re-operation was 99% after 1 year and 91% after 10 years (3).

Compared with other outcomes of the David procedure, such as short and mid-term survival, data on pre- and intra-operative risk factors for later re-operation are less well characterized (4, 5).

Various risk factors for valve failure after the David procedure have been described. These include specific morphological substrates such as bicuspid aortic valve, insufficient coaptation height or residual cusp prolapse, as well as surgeon's experience (2, 5–8).

In addition to morphological substrates and surgical experience, emergency David procedures might also carry a higher risk for later re-operation. However, David procedure in acute type A aortic dissection (AADA) has not been described as an independent risk factor for valve failure yet, although it is generally considered more demanding.

Against this background, the present study aims to systematically analyze these determinants of reoperation based on a large two-center cohort from experienced aortic programmes. In order to facilitate individualized risk stratification for future patients and to refine evidence-informed decision-making in valve-sparing root surgery, we additionally translate these determinants into an individual risk score for re-operation in David patients.

## 2 Material and methods

Study design was a retrospective cohort study with data from two European tertiary care institutions. Ethical approval was granted by local ethics committees (3552-2017 and 2025/Disp/AOUBo). Due to the retrospective nature of the analysis, individual patient consent was not needed and therefore waived. Available retrospective data from patients undergoing the David procedure in both institutions were merged to create one dataset for analysis and model development.

Transparent reporting of a multivariable prediction model for individual prognosis or diagnosis (TRIPOD) was used for model reporting and structuring this article.

The analysis performed resembles type 1b (i.e., development of a prediction model on a whole dataset, followed by internal assessment of calibration and discrimination) (9).

All available corresponding parameters were included. Patients underwent primary operation between 07/1993 and 01/2025. Median available follow up time was 120 months (IQR: 141 months).

Outcome assessed in the prediction analysis was re-operation after David operation. Available pre- and intra-operative data were assessed as potential predictors.

Sample size resembles all retrospectively available electronic patient records in the databases of patients undergoing David operation in both participating institutions. Missing data was handled through listwise deletion (i.e., complete case analysis).

Statistics were performed using Jamovi Desktop Version 2.6.44 for Linux, a statistic software based on R (10, 11). Descriptive statistics (median, IQR, minimum, maximum) and frequencies (*n*, %) were performed. Normality of continuous variables was assessed using the Shapiro–Wilk test (*W*, *p*). The influence of pre- and intra-operative variables on re-operation was tested using binomial univariable logistic regression analysis. In order to obtain a parsimonious model, variables with *p*-values <0.1 were investigated in binomial multivariable logistic regression analysis (12). In multivariable logistic regression analyses, variance was assessed using Nagelkerke's pseudo-*R*^2^. Collinearity statistics, including variance inflation factor (VIF) and tolerance were performed to ensure that multicollinearity did not play a role in the model.

Variables with a significance level of *p* < 0.05 were examined to create a prediction model. A multivariable logistic regression analysis with these variables was conducted for model fitting. Bootstrapping (bias-corrected and accelerated, BCa) with 1,000 resamples allowed stability assessment of 95% confidence intervals for the odds ratios of the final model (13, 14). For score creation, the intercept and estimates (B) were used in a linear prediction function (Score = Intercept + (B1 × variable 1) + (B*n* × variable *n*) + …). Logistic transformation was applied to calculate individual event probability (*p* = 1/(1 + *e*^(−Score)^).

Based on calculated event probabilities a receiver operating characteristic (ROC) analysis was conducted for score discrimination analysis (15, 16). Youden Index (J) was used to identify the optimal cutoff value. For calibration analysis, calibration in the large and slope were performed on the original score values.

We chose a binary reoperation endpoint (“reoperation at any time”) to facilitate development of a multivariable risk prediction model that does not rely on proportional hazards assumptions or on estimation of baseline survival for absolute risk computation. In addition, defining fixed time-point endpoints (e.g., 5/10/15 years) would have reduced the number of analyzable events within each time window and increased model instability given heterogeneous follow-up. To assess the robustness of our model and to account for different follow-up times, we performed a sensitivity analysis. To this end, we used a multivariable Cox proportional hazards regression analysis. The analysis included all variables of our final model. To assess potential effects of the surgical era, an additional Cox regression including the years since baseline (1993) was performed.

## 3 Results

1,074 patients, who underwent David operation for aortic valve reconstruction, were included in this study. Median age at operation was 54 years (IQR: 22). 74.9% (*n* = 804) were male. 128 patients had a bicuspid aortic valve (11.9%). In 310 cases (28.9%) a valsalva prosthesis was used. Marfan syndrome was present in 17.6% of patients (*n* = 189). 16.3% of all cases were urgent or emergent cases (*n* = 175). Median pre-operative left ventricular ejection fraction (LVEF) was 60% (IQR: 10). Median cardio-pulmonary-bypass-time (CPB-time) was 179 min (IQR: 73). In hospital mortality was 3.7% (*n* = 40). 130 patients (12.2%) had to be re-operated on after a median time of 4.43 years (IQR: 7.87). Detailed patient characteristics (descriptive statistics and frequencies) can be found in Tables 1, 2.

**Table 1:** Descriptive statistics of pre- and intraoperative variables including Shapiro Wilk test for normality.

Variable	Median	IQR	Min	Max	Shapiro–Wilk *W*	Shapiro–Wilk *p*
Age at operation [y]	54.00	22	8	83	0.9691	<.001
Height [cm]	178.00	15	144	213	0.9941	<.001
Weight [kg]	82.00	20	35	162	0.9837	<.001
BMI	25.95	5	13.8	46.9	0.9816	<.001
BSA	2.01	311	1.2	2.94	0.9959	0.006
Preoperative LVEF [%]	60.00	10	19	87	0.9623	<.001
CPB-time [min]	179	73	58	509	0.8938	<.001
X-clamp-time [min]	130	47	37	347	0.9431	<.001
Circulatory-arrest-time [min]	0.00	15	0	216	0.5909	<.001
Antegrade-cerebral-perfusion-time [min]	0	8	0	254	0.4800	<.001
Prosthesis size [mm]	28	2	20	34	0.8917	<.001

BMI, body mass index; BSA, body surface area; LVEF, left ventricular ejection fraction; CPB, cardio-pulmonary-bypass.

**Table 2:** Frequencies of pre- and intra-operative variables, as well as outcome (re-operation) and in-hospital mortality.

Variable	*n*	%
Male sex	804	74.9%
Pre-operative AI grade		
0	99	9.7%
1	221	21.7%
2	591	58.1%
3	107	10.5%
Emergency	175	16.3%
AADA	171	16.0%
CADA	38	3.5%
Root or bulbus aneurysm	584	54.6%
Ascending aneurysm	744	69.5%
Arch aneurysm	153	14.3%
Descending Aneurysm	64	6.0%
Arterial hypertension	664	62.2%
Hyperlipidemia	261	24.5%
Diabetes mellitus	49	4.6%
Smoking	309	28.9%
COPD	58	5.4%
Marfan syndrome	189	17.6%
NYHA		
I	598	58.7%
II	268	26.3%
III	132	13.0%
IV	20	2.0%
Coronary artery disease	184	17.2%
Re-do	52	4.8%
Bicuspid aortic valve	128	11.9%
Concommitant procedure	603	56.2%
Cusp repair	193	18.3%
Valsalva prosthesis	310	28.9%
Reoperation	130	12.2%
In hospital mortality	40	3.7%

AI, aortic insufficiency; AADA, acute aortic dissection type A; CADA, chronic aortic dissection type A; COPD, chronic obstructive pulmonary disease; NYHA, classification of New York Heart Association.

In univariable binomial logistic regression analysis potential pre- and intra-operative predictors of re-operation (*p* < 0.1) were identified as age at operation (OR: 0.98; 95% CI: 0.97–0.99; *p* < 0.001), male sex (OR: 1.56; 95% CI: 0.98–2.49; *p* = 0.061), pre-operative aortic-insufficiency (AI) grade (OR: 1.61; 95% CI: 1.23–2.1; *p* < 0.001), pre-operative left ventricular ejection fraction (LVEF) (OR: 0.98; 95% CI: 0.96–0.996; *p* = 0.019), arterial hypertension (OR: 0.65; 95% CI: 0.45–0.94; *p* = 0,024), diabetes mellitus (OR: 0.29; 95% CI: 0.07–1.23; *p* = 0,093), New York Heart Association grade (NYHA) (OR: 1.35; 95% CI: 1.09–1.69; *p* = 0.007), bicuspid aortic valve (OR: 1.97; 95% CI: 1.21–3.19; *p* = 0.006), cardio-pulmonary-bypass-time (OR: 0.996; 95% CI: 0.993–0.999; *p* = 0.022), antegrade-cerebral-perfusion-time (OR: 0.99; 95% CI: 0.98–0.998; *p* = 0.017) and prosthesis size (OR: 0.79; 95% CI: 0.71–0.88; *p* < 0.001). Table 3 shows the detailed results of the univariable logistic regression analysis of all investigated variables.

**Table 3:** Univariable binomial logistic regression showing the influence of induvidual pre- and intra-operative parameters on re-operation in David patients.

Variable	Odds-ratio	95% confidence interval		*p*
		Lower	Upper	
Age at operation [y]	0.979	0.968	0.991	**<**.**001**
Male sex	1.5614	0.9795	2.489	**0**.**061**
Height [cm]	1.0047	0.98737	1.02	0.6
Weight [kg]	0.995	0.9835	1.006	0.375
Pre-operative AI grade	1.6078	1.2337	2.095	**<**.**001**
Preoperative LVEF [%]	0.977	0.958	0.996	**0**.**019**
Emergency	0.977	0.594	1.608	0.927
AADA	1.008	0.612	1.66	0.974
CADA	0.39	0.0928	1.64	0.199
Root or bulbus aneurysm	0.908	0.629	1.311	0.606
Ascending aneurysm	1.164	0.7737	1.751	0.466
Arch aneurysm	0.696	0.389	1.247	0.223
Descending Aneurysm	0.876	0.391	1.963	0.747
Arterial hypertension	0.653	0.451	0.944	**0**.**024**
Hyperlipidemia	1.272	0.845	1.917	0.249
Diabetes mellitus	0.294	0.0706	1.226	**0**.**093**
Smoking	0.859	0.567	1.303	0.475
COPD	1.004	0.445	2.264	0.993
Marfan syndrome	0.886	0.539	1.455	0.633
NYHA	1.3543	1.0859	1.689	**0**.**007**
Coronary artery disease	0.639	0.369	1.107	0.11
Re-do	0.758	0.296	1.943	0.564
Bicuspid aortic valve	1.971	1.216	3.193	**0**.**006**
CPB-time [min]	0.996	0.993	0.999	**0**.**022**
X-clamp-time [min]	0.997	0.992	1.002	0.227
Circulatory-arrest-time [min]	0.998	0.99	1.007	0.696
Antegrade-cerebral-perfusion-time [min]	0.988	0.979	0.998	**0**.**017**
Concommitant procedure	0.957	0.662	1.385	0.817
Cusp repair	1.096	0.687	1.75	0.7
Prosthesis size [mm]	0.794	0.714	0.882	**<**.**001**
Valsalva prosthesis	0.851	0.562	1.29	0.448

AI, aortic insufficiency; LVEF, left ventricular ejection fraction; AADA, acute aortic dissection type A; CADA, chronic aortic dissection type A; COPD, chronic obstructive pulmonary disease; NYHA, classification of New York Heart Association; CPB, cardio-pulmonary-bypass.

*P*-values < 0.1 are printed in bold.

After listwise deletion, data of 952 patients could be included in the multivariable binomial logistic regression analysis of the potential predictors.

Multicollinearity did not play a substantial role, as the variance inflation factor (VIF) was below 2 and tolerance was 0.7 and above. A modest predictive power was indicated through a Nagelkerke's *R*^2^ of 0.140. However, male sex (OR: 1.98; 95% CI: 1.16– 3.39, *p* = 0.012), pre-operative AI grade (OR: 1.61; 95% CI: 1.21–2.14; *p* = 0.001), NYHA grade (OR: 1.29; 95% CI: 1.0043–1.67; *p* = 0.046) and a bicuspid aortic valve (OR: 2.03, 95% CI: 1.18–3.49; *p* = 0.011) were associated with a higher risk, whereas a higher age at operation (OR: 0.98; 95% CI: 0.97–0.996; *p* = 0.011) and larger prosthesis size (OR: 0.76; 95% CI: 0.66–0.86; *p* < 0.001) were associated with a lower risk of re-operation. Full results of the multivariable regression analysis can be found in Table 4.

**Table 4:** Multivariable binomial logistic regression showing the influence of pre- and intra-operative parameters on re-operation in David patients.

Variable	Estimate (B)	Standard error (SE)	Odds-ratio	95% confidence interval		*p*	VIF	Tolerance
				Lower	Upper			
Male sex	0.68527	0.27274	1.984	1.1627	3.387	**0**.**012**	1.13	0.881
Age at operation [y]	−0.01949	0.00765	0.981	0.9661	0.996	**0**.**011**	1.43	0.7
Pre-operative AI grade	0.47632	0.14517	1.610	1.2114	2.140	**0**.**001**	1.14	0.874
Preoperative LVEF [%]	−0.01763	0.01067	0.983	0.9622	1.003	0.099	1.06	0.943
Arterial hypertension	−0.28145	0.22713	0.755	0.4835	1.178	0.215	1.23	0.812
Diabetes mellitus	−0.84471	0.74890	0.43	0.0990	1.865	0.259	1.02	0.977
NYHA	0.25751	0.1292	1.294	1.0043	1.667	**0**.**046**	1.18	0.846
Bicuspid aortic valve	0.70628	0.27735	2.026	1.1767	3.49	**0**.**011**	1.09	0.916
CPB-time [min]	−0.000825	0.00205	0.999	0.9952	1.003	0.688	1.33	0.751
Antegrade-cerebral-perfusion-time [min]	−0.00755	0.00573	0.992	0.9814	1.004	0.187	1.33	0.75
Prosthesis size [mm]	−0.28064	0.06524	0.755	0.6646	0.858	**<**.**001**	1.16	0.861

AI, aortic insufficiency; LVEF, left ventricular ejection fraction; NYHA, classification of New York Heart Association; CPB, cardio-pulmonary-bypass. Nagelkerke's pseudo-*R*^2^ = 0.140; Intercept 6.59145, *n* = 952 (out of 1,074 patients).

*P*-values < 0.05 are printed in bold.

Model fitting of these predictors allowed the creation of the final prediciton model (results displayed in Table 5).

**Table 5:** Model fitting (multivariable regression analysis of independent predictors of re-operation in david patients), including bootstrapped (bias-corrected and accelerated, Bca, 1,000 resamples) 95% confidence interval of odds ratios.

Variable	Estimate (B)	Standard error (SE)	Odds-ratio	95% confidence interval (model fit)		95% confidence intervals (bootstrap Bca)		*p*	VIF	Tolerance
				Lower	Upper	Lower	Upper			
Male sex	0.668	0.26535	1.95	1.159	3.281	1.2858	3.728	0.012	1.13	0.886
Age at operation [y]	−0.0265	0.00694	0.974	0.961	0.987	0.9603	0.987	<.001	1.19	0.84
Pre-operative AI grade	0.5031	0.14346	1.654	1.248	2.191	1.3292	2.323	<.001	1.12	0.893
NYHA	0.2786	0.12623	1.321	1.032	1.692	1.0215	1.718	0.027	1.16	0.863
Bicuspid aortic valve	0.7623	0.27417	2.143	1.252	3.668	1.2824	3.926	0.005	1.09	0.921
Prosthesis size [mm]	−0.2749	0.06367	0.76	0.67	0.861	0.6634	0.862	<.001	1.16	0.861

AI, aortic insufficiency; LVEF, left ventricular ejection fraction; NYHA, classification of New York Heart Association. Nagelkerke's pseudo-*R*^2^ = 0.121; Intercept 5.2278, *n* = 993 (out of 1,074 patients).

The following linear prediction function was used to generate the pre and intra-operative risk score for re-operation of David patients (PRO-David) − Score (logit) = 5.2278 + (0.668 × male sex) + (−0.0265 × age at operation [y]) + (0.5031 × pre-operative AI grade) + (0.2786 × NYHA) + (0.7623 × bicuspid aortic valve) + (−0.2749 × prosthesis size [mm]). Similar bootstrapped odds ratio 95% confidence intervals indicated stability of the final model (Table 5).

**Figure 1:**
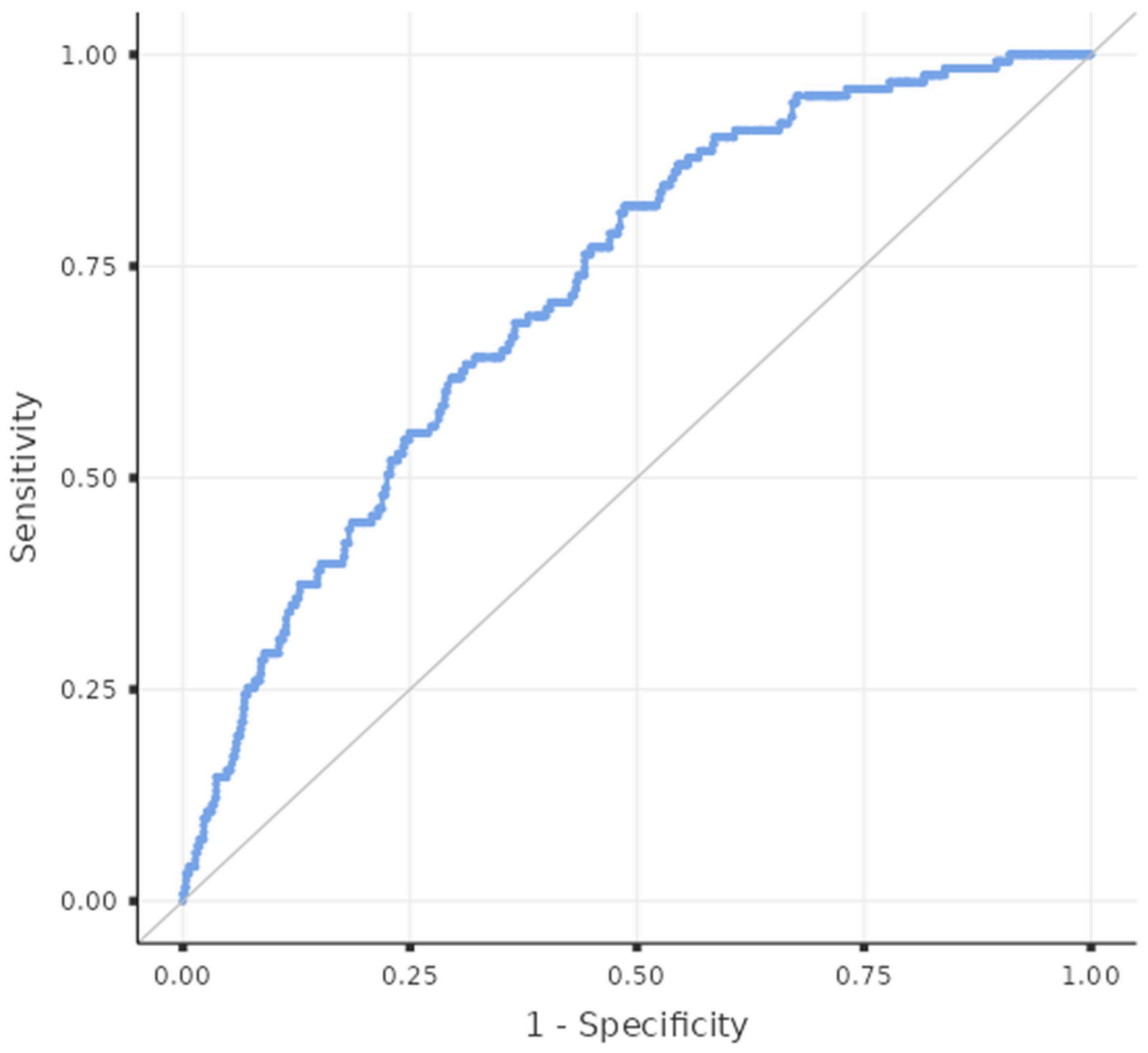
Receiver operating characteristic (ROC) analysis based on calculated event probabilities of the score, area under the curve (AUC) 0.722 (95% CI: 0.677–0.766).

ROC analysis based on calculated event probabilities, showed a moderate discriminatory ability of the score with an area under the curve (AUC) of 0.722 (95% CI: 0.677–0.766). The ROC curve is depicted in Figure 1. Maximum Youden Index (J) was 0.335 at a cut-off value of 0.095 (sensitivity: 82.1%, specificity: 51.4%). Calibration analysis (based on the original score values as the predictor of re-operation), provided a calibration intercept (calibration-in-the-large) of −0.00 (95% CI: −0.49–0.49) and a calibration slope of 1.00 (95% CI: 0.75–1.26). These values indicate a well calibrated model in the original cohort.

Multivariable Cox regression, including the parameters of our final model, showed that all variables, except NYHA class, remained significant when taking different follow up times into account (Table 6). When including years since baseline, surgical era did not show a statistically significant effect on re-operation (HR: 0.99; 95% CI: 0.96–1.03 *p* = 0.679). Results of other predictors remained stable.

**Table 6:** Sensitivity analysis using a multivariable Cox proportional hazards regression including all predictors of the final model.

Variable	Estimate (*B*)	Standard error (SE)	Hazard-ratio	95% confidence interval		*p*
				Lower	Upper	
Male sex	0.5404	0.2440	1.717	1.064	2.769	0.027
Age at operation [y]	−0.01625	0.006224	0.9839	0.9720	0.9960	0.009
Pre-operative AI grade	0.4501	0.1307	1.568	1.214	2.026	<.001
NYHA	0.1479	0.1143	1.159	0.9266	1.451	0.196
Bicuspid aortic valve	0.6851	0.2346	1.984	1.253	3.142	0.003
Prosthesis size [mm]	−0.1684	0.06089	0.8450	0.7500	0.9521	0.006

AI, aortic insufficiency; LVEF, left ventricular ejection fraction; NYHA, classification of New York Heart Association. *n* = 987 (out of 1,074 patients).

## 4 Discussion

While prior studies have identified single risk factors for re-operation after the David procedure, we focused on a characterization of the data towards an individual risk prediction model. In our cohort of patients, valve sparing root replacement was performed liberally with 16.3% emergency cases, 11.9% biscuspid valves and 18.3% cusp repair (Table 2). In addition, the cohort resembles the learning curve of many different surgeons. All these factors most likely led to a higher rate of re-operation of 12.2%.

Specific valve-related factors have been previously detected as risk factors for re-operation in David patients:

Patlolla et al. published a long term follow-up study of 342 patients who underwent valve sparing aortic root replacement at the Mayo Clinic from 1994 to 2017. They managed to identify larger pre-operative annulus diameters as a risk factor for re-operation (17). Recently, bicuspid valve and leaflet repair were reported as long-term factors associated with re-intervention (5, 18).

A long-term follow-up study of 300 patients found that preoperative aortic insufficiency ≥2 and a ventriculoaortic junction ≥29 mm were risk factors for late postoperative aortic insufficiency ≥2 (19).

In a multivariable approach, the level of coaptation within the graft was identified to be linked to the development of aortic insufficiency (6). Graft size mismatch (≥4 mm) and untreated cusp prolapse are also among reported valve-related risk factors for re-operation (7).

In our patient group, cusp repair was not an independent predictor of re-operation (*p* = 0.7).

In line with these data we identified pre-operative aortic insufficiency grade and a bicuspid valve morphology as risk factors for re-operation in our cohort of patients. These factors most likely reflect severity of morphological alterations of the aortic valve. Due to a lack of detailed preoperative data on valve morphology, we were, however, not able to review potential underlying mechanisms of these surrogate parameters. For example, there is a marked heterogeneity of BAV morphology that translates into different long term results of BAV repair. Further research should concentrate on distinct morphological aspects of valvular morphology.

Hyperlipidaemia and preoperative left ventricular ejection fraction were among the general preoperative risk factors previously identified, that could not be echoed in our analysis (2).

According to a single center study of 582 patients, another important predictor of re-operation was surgical experience. The study showed that the surgeon's experience had both, an impact on the peri-operative outcome and the long-term performance of the aortic valve (20). Experience of the surgeon was not investigated in this two center trial. Experience may also influence the surgical approach. Aspects of surgical technique (e.g., graft sizing, aggressiveness regarding cusp or commissure repair) have been described as possible risk factors for long term valve failure after aortic valve repair (7). In line with these findings our study found prosthetic size to be a relevant prognostic technical factor. Over time our policy of sizing has moved away from a policy to reduce anulus size towards a policy of using larger prosthesis (anulus size + 2–4 mm). This might reflect a more physiological approach towards valve function and possible excess of tissue. Further research is needed to clarify this notion.

Given that long-term durability after the David procedure is closely linked to surgeon experience and specific technical aspects, structured, dedicated training pathways and the implementation of a formalized “co-pilot” policy, whereby less experienced surgeons perform the operation under direct supervision of an expert during the learning curve should be taken into account.

In our study, NYHA grade was identified as an independent predictor of re-operation. NYHA grade had been reported as a predictor of re-operation in David patients before (21). Nonetheless, this parameter may be too general for a future refined prediction score. As NYHA grade is a parameter associated with complications and mortality after cardiac surgery (22), a relation to higher rates of re-operation seems plausible when interpreted as a marker of advanced disease stage (i.e., progressed ventricular remodelling as a result of long-term AI leading to higher operative risk). In multivariable Cox regression analysis, NYHA class did not remain statistically significant. This indicates that NYHA class may be a less stable parameter when taking different follow-up times into account.

Overall, Sensitivity analysis, using Cox regression, underlined the stability of our findings. This is in accordance with the observation that surgical era was not significantly associated with re-operation. Robustness of our final multivariable model was additionally supported by an events per variable (EPV) of >20.

## 5 Conclusion

The developed model and PRO-David score provide a promising first approach to identify patients at higher risk of re-operation after David procedure. Identified patients may then undergo a closer follow-up. In conclusion, it is essential to acknowledge that modes of valve failure after the David procedure can largely be grouped into two principal domains: intrinsic valvular morphological characteristics (such as bicuspid configuration, cusp prolapse, asymmetry, and geometric alterations affecting coaptation) and technical surgical factors (including graft sizing, reconstruction strategy, and overall surgical proficiency). Prospective risk stratification of individual patients should systematically incorporate both dimensions to better estimate long-term durability. Importantly, recognition of the strong influence of surgical expertise mandates structured training concepts and supervised implementation of the procedure during the learning curve. Beyond procedure-related aspects, shared decision-making must also integrate the individual patient's perspective when weighing valve-sparing surgery against alternative strategies such as mechanical or biological composite root replacement. As patient-centred outcome evaluation becomes increasingly relevant, these considerations—including anatomical risk profile, technical feasibility, and surgeon-specific expertise—should be explicitly reflected in contemporary clinical decision-making frameworks.

## 6 Limitations/future projects

This work represents a two-center retrospective cohort study. Center-specific influences can therefore not be ruled out. The developed risk model uses a binary re-operation endpoint (“re-operation at any time”). Therefore a differentiation between parameters leading to “early” in comparison to “late” reoperation cannot be made. Predictor selection was based on univariable *p*-values. This can potentially lead to instability. However, the approach helped to create a parsimonious model. Our prediction model is based on a single dataset. External validation is needed prior to a broader applicability. Nagelkerke's *R*^2^ of 0.140 indicates that other risk factors, not included in our analysis, may also impact the outcome “re-operation”. Despite quite a large number of patients, generalizations should hence be drawn with caution. Additionally, detailed analysis of geometric data of the valve such as leaflet length, valve symmetry and regurgitation characteristics, as well as detailed echocardiographic data remains to be analyzed in future studies to further refine the prediction model.

## Data Availability

The original contributions presented in the study are included in the article/Supplementary Material, further inquiries can be directed to the corresponding author.
